# Supplementary material for: Adaptation and validation of the Spanish version of the Being a Mother scale
Source: PeerJ. 2024 Oct 8;12:e18015. doi: 10.7717/peerj.18015 (PMC11468896; doi:10.7717/peerj.18015)
Supplement: Supplemental Information 1 — IQR: Interquartile range. [file peerj-12-18015-s001.docx]

| Supplementary Table 1  Descriptive characteristics of the items. | | | | |
| --- | --- | --- | --- | --- |
| **Item** | **Median (IQR)** | **Skewness** | **Cronbach's Alpha if Item Deleted** | **Spearman Correlation coefficient (r)** |
| 1. I have (not) felt confident about taking care my child.* | 0 (0-0) | 2.555 | 0.763 | 0.441 |
| 2. I have missed the life I had before I became pregnant with this child. | 1 (0-2) | 0.357 | 0.754 | 0.617 |
| 3. I have found it hard to manage the situation when my child cries. | 1 (0-2) | 0.461 | 0.750 | 0.661 |
| 4. I have (not) felt close to my child.* | 0 (0-0) | 6.698 | 0.779 | 0.227 |
| 5. I have felt lonely or isolated. | 0 (0-1) | 0.943 | 0.547 | 0.792 |
| 6. I have felt bored. | 0 (0-1) | 1.298 | 0.726 | 0.585 |
| 7. I have felt unsupported. | 0 (0-1) | 0.902 | 0.561 | 0.793 |
| 8. I have (not) felt alright about asking people for help or advice when I needed to.* | 0 (0-1) | 1.141 | 0.698 | 0.619 |
| 9. I have felt nervous or uneasy around my child. | 0 (0-1) | 0.639 | 0.728 | 0.707 |
| 10. I have been worried that something would. happen to my child. | 2 (1-2) | -0.540 | 0.772 | 0.534 |
| 11. I have been annoyed or irritated with my child. | 0 (0-1) | 0.822 | 0.743 | 0.629 |
| 12. I worry I am not as good as other mothers. | 0 (0-1) | 0.795 | 0.739 | 0.665 |
| 13. I have felt guilty. | 0 (0-1) | 0.874 | 0.730 | 0.692 |
| IQR: Interquartile range. |  |  |  |  |
