# Supplementary figures and images for: Adaptation and validation of the Spanish version of the Being a Mother scale
Source: PeerJ. 2024 Oct 8;12:e18015. doi: 10.7717/peerj.18015 (PMC11468896; doi:10.7717/peerj.18015)

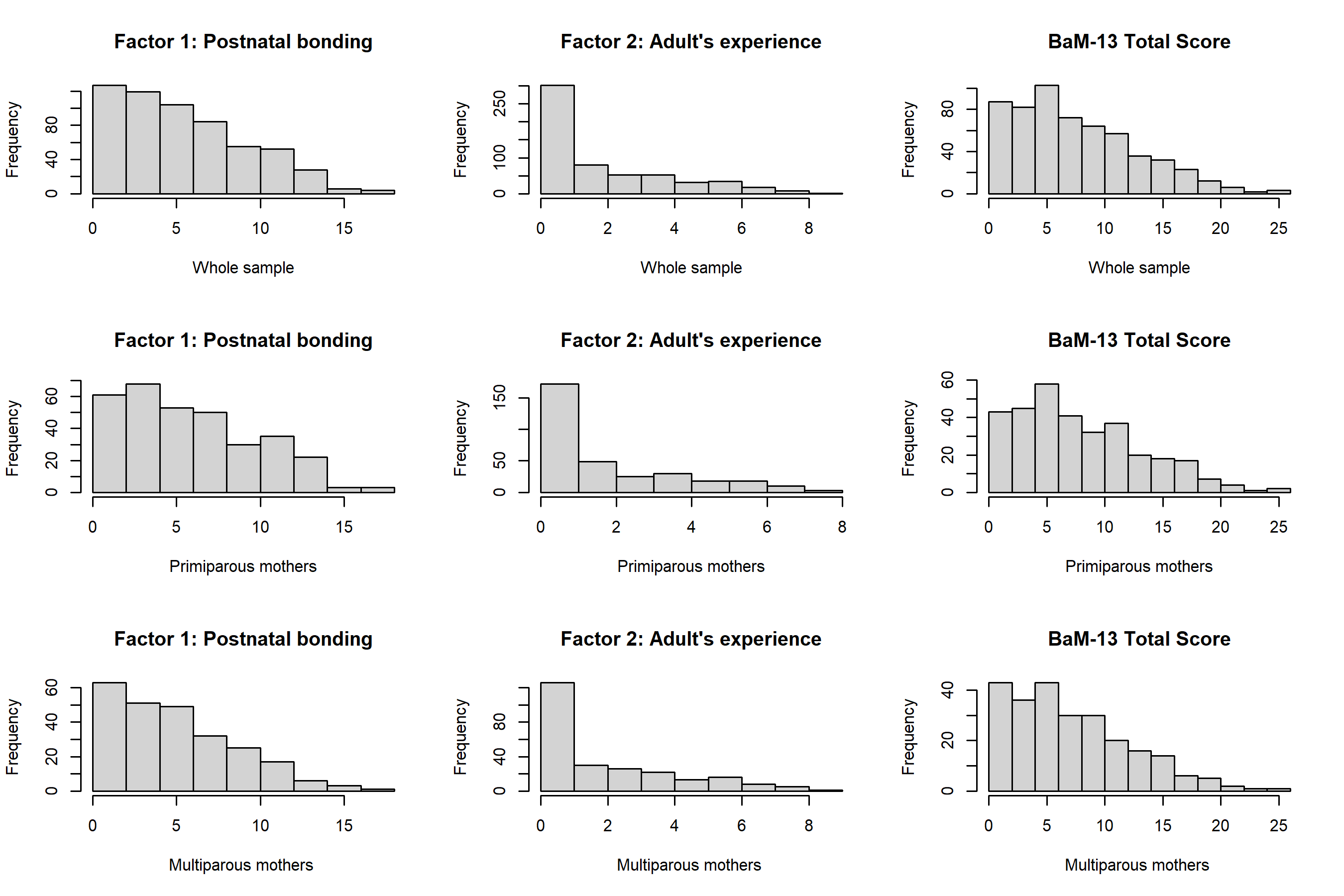

Supplement: Supplemental Information 3 [file peerj-12-18015-s003.tif]
