# Supplementary material for: Adaptation and validation of the Spanish version of the Being a Mother scale
Source: PeerJ. 2024 Oct 8;12:e18015. doi: 10.7717/peerj.18015 (PMC11468896; doi:10.7717/peerj.18015)
Supplement: Supplemental Information 4 [file peerj-12-18015-s004.pdf]

CONVERTIRSE EN MADRE (BaM-13)

Nombre:____ Fecha de hoy:____________________

Edad de su hijo/a: __________ (¿semanas o meses o años?)

¿Tiene usted más hijos? Si / No (en caso afirmativo, ¿qué edad tienen?:________________

Las preguntas más abajo nos ayudarán a comprender cómo está viviendo usted la experiencia de ser madre.

No hay respuestas correctas o incorrectas. Sólo son respuestas que nos indican cómo se ha estado sintiendo usted. Para cada pregunta, piense en cómo se ha estado sintiendo durante las últimas dos – tres semanas. Por favor, subraye una afirmación por cada pregunta.

<u>**Durante las últimas 2 - 3 semanas**</u>

1. Me he sentido segura cuidando a mi niño-a:
- ☐ Si, Siempre o casi siempre
- ☐ Si, a veces
- ☐ No, no muy a menudo
- ☐ No, casi nunca o nunca

2. He echado de menos la vida que tenía antes de quedarme embarazada de este niño-a:
- ☐ No, raramente o nunca
- ☐ No, no muy a menudo
- ☐ Si, algunas veces
- ☐ Si, siempre o casi siempre

3. Me ha resultado difícil manejar la situación cuando mi niño-a llora:
- ☐ No, raramente o nunca
- ☐ No, no muy a menudo
- ☐ Si, algunas veces
- ☐ Si, siempre o casi siempre

4. Me he sentido unido a mi niño-a:
- ☐ Si, siempre o casi siempre
- ☐ Si,algunas veces
- ☐ No, no muy a menudo
- ☐ No, raramente o nunca

5. Me he sentido sola o aislada:
- ☐ No, raramente o nunca
- ☐ No, no muy a menudo
- ☐ Si, algunas veces
- ☐ Si, siempre o casi siempre

Continúa en la siguiente página………

6. Me he sentido aburrida:

- ☐ No, raramente o nunca
- ☐ No, no muy a menudo
- ☐ Si, algunas veces
- ☐ Si, siempre o casi siempre

7. Me he sentido sin apoyo:

- ☐ No, raramente o nunca
- ☐ No, no muy a menudo
- ☐ Si, algunas veces
- ☐ Si, siempre o casi siempre

8. Me he sentido bien al pedir ayuda o consejo a la gente cuando lo he necesitado:

- ☐ Si, siempre o casi siempre
- ☐ Si,algunas veces
- ☐ No, no muy a menudo
- ☐ No, raramente o nunca

9. Me he sentido nerviosa o insegura con mi niño-a:

- ☐ No, raramente o nunca
- ☐ No, no muy a menudo
- ☐ Si, algunas veces
- ☐ Si, siempre o casi siempre

10. He estado preocupada por si le pasara algo a mi niño-a:

- ☐ No, raramente o nunca
- ☐ No, no muy a menudo
- ☐ Si, algunas veces
- ☐ Si, siempre o casi siempre

11. He estado enfadada o irritada con mi niño-a:

- ☐ No, raramente o nunca
- ☐ No, no muy a menudo
- ☐ Si, algunas veces
- ☐ Si, siempre o casi siempre

12. Me preocupa no ser tan buena como otras madres:

- ☐ No, raramente o nunca
- ☐ No, no muy a menudo
- ☐ Si, algunas veces
- ☐ Si, siempre o casi siempre

13. Me he sentido culpable:

- ☐ No, raramente o nunca
- ☐ No, no muy a menudo
- ☐ Si, algunas veces
- ☐ Si, siempre o casi siempre

En caso de que a usted la experiencia de ser madre le haya resultado muy estresante, muy difícil, o desagradable, ¿por qué motivo piensa usted que ha sido así?:_____________________

____

____
